# Bioactive Composition and Antioxidant and Glycemic‐Regulating Properties of *Basella alba* L. Leaf, Fruit, and Stem: GC–MS, FTIR, In Vitro and In Silico Insights

**DOI:** 10.1002/fsn3.71936

**Published:** 2026-06-17

**Authors:** Shorna Das, Satyajit Roy Rony, Bidduth Kumar Sarkar, Sukalyan Kumar Kundu, Pradip Debnath

**Affiliations:** ^1^ Natural Products Research Laboratory, Department of Pharmacy, Faculty of Science Comilla University Cumilla Bangladesh; ^2^ Pharmaceutical Sciences Research Division Bangladesh Council of Scientific and Industrial Research (BCSIR) Dhaka Bangladesh; ^3^ Department of Pharmacy Jahangirnagar University Savar Dhaka Bangladesh

**Keywords:** anti‐diabetic, antioxidant, *Basella alba*, FTIR, GC–MS, in silico study

## Abstract

*Basella alba* L. (Malabar spinach/Indian spinach) is a widely consumed leafy vegetable with traditional medicinal uses. This study aimed to comparatively evaluate the phytochemical composition, functional groups, bioactive constituents, molecular docking and in vitro biological activities of leaf, fruit, and stem extracts of *Basella alba* . Quantitative results indicated that both TPC and TFC followed the order fruit > leaf > stem, with values of 654.18 ± 7.97, 394.19 ± 4.60, 335.97 ± 6.26 mg GAE/g and 720.36 ± 13.99, 684.71 ± 16.46, 561.29 ± 5.93 mg QE/g, respectively (*p* < 0.05). FTIR analysis identified functional groups such as O–H, C=C, C–N, C=O corresponding to alcohols, amines, esters, carboxylic acids etc. consistent with GC–MS profiles. GC–MS analysis identified some top major compounds, including 1‐methyl‐5‐fluorouracil (9.63%), 3‐methylquinoline (8.55%), 1‐ethyl‐1H‐pyrazole‐3,4‐diamine (6.83%) in leaf, fruit and stem extract respectively. Antioxidant assays showed that the fruit extract exhibited the strongest activity, with the lowest IC₅₀ values for DPPH (25.27 ± 1.41 μg/mL) and H_2_O_2_ (44.67 ± 2.54 μg/mL), and the highest TAC (570.09 ± 27.27 mg AAE/g), followed by leaf and stem extracts (*p* < 0.05), In contrast, the leaf extract exhibited the highest antidiabetic activity with IC_50_ = 50.63 ± 1.93 μg/mL (α‐amylase) and 27.81 ± 1.85 μg/mL (α‐glucosidase), followed by fruit and stem extracts (*p* < 0.05). Molecular docking indicated that 3‐methylquinoline from the fruit has the highest binding affinity for oxidoreductase (−8.3 kcal/mol), whereas 1H‐naphth[1,2‐d]imidazole from the leaf showed the strongest affinities for α‐amylase (−6.6 kcal/mol) and maltase‐glucoamylase (−7.1 kcal/mol), with favorable drug‐like properties. These findings support the ethnomedicinal relevence of *B. alba* and suggest its different parts may provide complementary therapeutic benefits.

## Graphical abstract

The bioactive composition, antioxidant capacity, and glycemic‐regulating properties of *Basella alba* L. leaf, fruit, and stem were comparatively analyzed using gas chromatography–mass spectrometry (GC–MS), Fourier‐transform infrared spectroscopy (FTIR), in vitro assays, and molecular docking analyses. The fruit extract demonstrated the highest phenolic and flavonoid contents as well as the most pronounced antioxidant activity. In contrast, the leaf extract exhibited the strongest inhibitory effects on α‐amylase and α‐glucosidase. GC–MS profiling revealed a range of bioactive compounds with favorable binding affinities for antioxidant and antidiabetic targets, thereby supporting the ethnomedicinal significance and therapeutic potential of various *B. alba* plant parts.

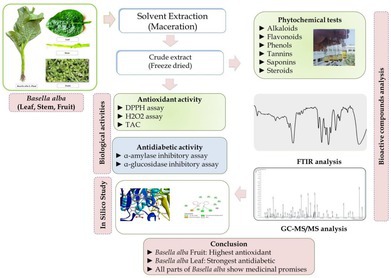

## 1 Introduction

Nature serves as a vast reservoir of medicinal plants containing diverse secondary metabolites, commonly referred to as phytochemicals, which have been utilized in traditional medicine systems for a long time (Ouandaogo et al. 2023). *Basella alba* L., commonly known as Malabar spinach or Ceylon spinach, is a soft‐stemmed, fast‐growing perennial vine belonging to the family Basellaceae. It is a widely consumed, green, leafy vegetable, particularly popular in tropical and subtropical regions, such as Africa and Asia. Traditionally, *B. alba* has been used to treat constipation, digestive disorders, skin diseases, and inflammation. In Indian and Chinese folk medicine, it is valued for its antimicrobial, diuretic, anti‐inflammatory, and detoxifying properties (Bamidele et al. 2025; Halayal et al. 2025; Zhang, Shuai, et al. 2024).

Scientific exploration of *B. alba* has expanded in recent years due to its rich repository of bioactive constituents, including polyphenols, flavonoids, alkaloids, tannins, saponins, glycosides, and steroids (Dahanayaka et al. 2025). These phytochemicals have been associated with antibacterial, anti‐hyperglycemic, antioxidant, anti‐inflammatory, cytotoxic, and anti‐proliferative activities, as demonstrated by in vitro, preclinical, and clinical investigations (Nur et al. 2023). However, most previous studies have focused either on a single plant part or on one or two pharmacological activities, leaving a gap in comprehensive comparative evaluations.

In this study, Phytochemical profiling of *B. alba* leaf, fruit, and stem was conducted through qualitative phytochemical assessment, quantitative evaluation of total phenol and flavonoids followed by spectral analysis, for example, FTIR and GC–MS. The combined application of FTIR and GC–MS provides complementary information: FTIR confirms the presence of specific functional groups, whereas GC–MS identifies the exact bioactive compounds present (Ella Nkogo et al. 2024). Such an integrated approach enhances the reliability of phytochemical profiling.

Oxidative stress, caused by an imbalance between reactive oxygen/nitrogen species (ROS/RNS) and endogenous antioxidants, disrupts cellular redox homeostasis (Pisoschi and Pop 2015). This imbalance contributes to the pathogenesis of several chronic diseases, including diabetes mellitus, neurodegenerative disorders, chronic inflammation, and cancer. Plant‐derived antioxidants, such as polyphenols, flavonoids, and tannins, are capable of neutralizing ROS and RNS, thereby mitigating oxidative stress–induced cellular damage (Marjanovic et al. 2021). Diabetes mellitus, one of the most prevalent chronic metabolic disorders worldwide, is characterized by persistent hyperglycemia and associated complications (Shaw et al. 2010; Bamagous et al. 2018). Although conventional treatments such as hypoglycemic drugs, diet and exercise helps regulate blood glucose, they do not provide a definitive cure. Given the established role of oxidative stress in diabetes progression, natural antioxidants from plants, such as *B. alba* , are being extensively explored as alternative therapeutic agents (Bamagous et al. 2018). The present study provides a comprehensive comparative evaluation of the leaf, stem, and fruit of *B. alba* through qualitative and quantitative phytochemical screening, FTIR, and GC–MS analyses with in vitro antioxidant and anti‐diabetic assays. Major compounds identified by GC–MS were further assessed via molecular docking and ADME/T analysis. Molecular docking is an integral part of computer aided drug design to identify potential lead compounds. This approach is essential in drug discovery and development, as it helps analyze how a drug interacts with its receptor and predicts the binding affinity and activity of a ligand at the target protein's active site (Al‐rajhi et al. 2023; Qanash et al. 2023) The antioxidant potential of each part of this plant was evaluated using DPPH, H_2_O_2_ scavenging, and total antioxidant capacity (TAC) assays. At the same time, anti‐hyperglycemic activity was assessed through in vitro inhibition of carbohydrate‐hydrolyzing enzymes, including α‐amylase and α‐glucosidase.

As previous studies lack comparative assessments (Bamidele et al. 2025; Halayal et al. 2025; Kumar et al. 2023; Nur et al. 2023) of phytochemical profiling and bioactivity evaluation of various parts of *Basella alba* , the present integrated approach aims to identify the most pharmacologically active part of *B. alba* through comparative evaluation, thereby providing evidence‐based validation of its traditional ethno‐medicinal use and highlighting its potential for drug development.

## 2 Materials and Methods

### 2.1 Chemicals and Reagents

Methanol (Merck, India), 2,2‐diphenyl‐1‐picrylhydrazyl (DPPH), Folin–Ciocalteu reagent, ascorbic acid, 3,5‐dinitrosalicylic acid (DNS), hydrogen peroxide, ferric chloride, sulfuric acid, sodium phosphate, ammonium molybdate, and phosphate buffer were procured from Sigma‐Aldrich (Germany). All chemicals and reagents used were of analytical grade. Distilled water was used throughout the experiments.

### 2.2 Collection and Identification of Plant

Fresh aerial parts of *B. alba* plants were procured from the village of Telinagar, Brahmanbaria (between 23°39′ N and 24°16′ N latitude and 90°44′ E and 91°51′ E longitude), Bangladesh, October 2024. Fruits of the same species were collected from the same source to ensure taxonomic uniformity and consistency of the cultivation site in December 2024. The fresh leaves, stems, and raw fruits of this plant were washed with tap water initially then rinsed in deionized water. After that, all parts were air dried for 2 weeks in order to remove excess water content, at room temperature under shed to prevent direct sunlight.

### 2.3 Preparation of Plant Material

The dried samples were coarsely powdered using a laboratory grinder (Labtech, China). The powdered plant materials were stored in airtight glass containers, protected from light and moisture, until further analysis was conducted. The powder 200 g of each sample was macerated in 2000 mL of 80% methanol, into three separate containers for 15 days (Omotoso et al. 2024) with occasional stirring (Abou‐Taleb et al. 2025) to improve contact between solvent and plant materials, in order to extract the phytochemicals efficiently. At a later stage, the mixtures were first filtered using clean muslin fabric and after that with Whatman No. 1 filter paper. Then, each filtrate was condensed to produce a concentrated extract using a rotary evaporator (RE100‐Pro, DLAB USA) at 40°C and freeze dried at −40°C then stored in capped vials at 4°C prior to further experimental procedure (Baskaran et al. 2015).

### 2.4 Preliminary Phytochemical Screening

The phytochemical presence in the crude extracts of *B. alba* plant parts, that is, leaf, fruit, stem were detected using standard protocol as described by Shaikh and Patil (2020). The standard solution of leaf, fruit and stem extracts were prepared by dissolving 100 mg of each extract in 10 mL methanol. These solutions were then used for screening the presence of different phytochemicals.

### 2.5 Quantification of Total Phenol Content

The total phenolic content (TPC) of *B. alba* plant part extracts was determined using the Folin–Ciocalteu colorimetric method, following the procedure described by El Kamari et al. (2024) with slight modifications. Briefly, 0.5 mL of each extract was mixed with Folin–Ciocalteu reagent, followed by the addition of 2 mL of 7.5% sodium carbonate (Na_2_CO_3_) solution. The mixtures were incubated at 25°C for 30 min, after which the absorbance was measured at 760 nm using a UV–Vis spectrophotometer. A standard calibration curve was prepared using gallic acid at concentrations ranging from 125 to 1000 μg/mL. Results were expressed as milligrams of gallic acid equivalents per gram of dry weight (mg GAE/g DW). A blank solution, prepared under the same conditions without plant extract, was used for background correction.

### 2.6 Quantification of Total Flavonoid Content

The total flavonoid content (TFC) of *B. alba* plant part extracts was determined following the aluminum chloride colorimetric method as described by Saleem et al. (2025) with minor modifications. Briefly, 0.5 mL of each extract was mixed with 2.5 mL of distilled water, followed by the addition of 150 μL sodium nitrite (NaNO_2_) solution and incubation for 5 min. Subsequently, 1 mL of 10 mM sodium hydroxide (NaOH) and 0.55 mL of distilled water were added, thoroughly mixed, and then centrifuged at 4000 rpm for 10 min. The absorbance of each reaction mixture was measured at 510 nm using a UV–Vis spectrophotometer. A calibration curve was constructed using quercetin standard solutions, and the results were expressed as milligrams of quercetin equivalents per gram of dry weight (mg QE/g DW). A blank solution prepared under identical conditions, without the addition of plant extract, served as the background control.

### 2.7 FTIR Analysis

The functional groups present in *B. alba* plant part extracts were determined by Fourier‐transform infrared (FTIR) spectroscopy following the method described by Uddin et al. (2023) and Ulambayar et al. (2024), with minor modifications. Briefly, 10 mg of each lyophilized extract was thoroughly mixed with 100 mg of spectroscopic‐grade potassium bromide (KBr) to obtain a homogeneous mixture. The mixture was compressed into thin translucent discs using a hydraulic press for analysis. The prepared KBr pellets were placed into the sample holder of a PerkinElmer Spectrum 2000 infrared spectrometer (PerkinElmer Inc., MA, USA). Spectral acquisition and analysis were performed using Spectrum software version 10.03.09.0139 (PerkinElmer Inc., MA, USA). The FTIR spectra were recorded in the wavenumber range of 4000–400 cm^−1^ with a resolution of 4 cm^−1^ to identify the functional groups present in the extracts.

### 2.8 Gas Chromatography‐Triple Quadrupole Mass Spectrometry (GC–MS/MS) Analysis

GC–MS/MS analysis of *B. alba* extracts was carried out using a gas chromatograph (GC–MS/MS, Shimadzu, Japan) coupled with a triple quadrupole mass spectrometer (GC–MS/MS TQ 8040, Shimadzu, Japan). A Rxi‐5 ms fused silica capillary column (30 m in length, 0.25 μm in thickness, 0.25 μm in diameter) was used to separate bioactive compounds from the sample. Helium gas (pressure, 53.5 kPa; total flow rate, 11.0 mL/min; column flow rate, 1.0 mL/min) was used as the carrier gas. The following temperature conditions were applied to the chromatogram: injection temperature, 250°C; column temperature, 50°C; oven temperature, 500°C for 1 min, 200°C for 2 min, and then 300°C for 7 min. Also, the ion source temperature was 230°C, the interface temperature was 250°C, the solvent cut time was 3.5 min., the data acquisition mode was Q3 scan mode, and the m/z value was set for 50–600. The total runtime required to complete the GC‐QqQ (MS/MS) analysis was 39 min. The bioactive chemicals present in the extract were identified by comparing the mass spectra with the GC–MS data included in the U.S. National Institute of Science and Technology (NIST) and Wiley libraries, with their retention time obtained under the same conditions and comparing their MS fragmentation profile with the literature (Kim et al. 2016; Obaidullah et al. 2021).

### 2.9 Antioxidant Activity

Three different methods were utilized to evaluate the antioxidant activity of leaf, fruit, and stem extracts of *B. alba* .

#### 2.9.1 DPPH Assay

The free radical scavenging ability of the extracts was determined using the 2,2‐diphenyl‐1‐picrylhydrazyl (DPPH) assay, following the method described by Althaher et al. (2024) with minor modifications. Briefly, 0.5 mL of each extract solution (62.5–1000 μg/mL) was mixed with 2.5 mL of 0.1 mM methanolic DPPH solution. The mixture was vortexed for 15 s and incubated at room temperature in the dark for 30 min. Ascorbic acid was used as the positive control. After incubation, the absorbance of both standard and sample solutions was measured at 517 nm using a UV–Vis spectrophotometer. The percentage of DPPH radical scavenging activity was calculated using the formula:

$$\text{Scavenging activity}\;\left(\%\right)=\frac{\text{Absorbance of control}-\text{Absorbance of sample}}{\text{Absorbance of control}}\times 100$$

#### 2.9.2 Hydrogen Peroxide (H_2_O_2_ ) Scavenging Assay

Hydrogen peroxide scavenging activity was evaluated according to the method described by Adjimani and Asare (2015). A 40 mM H_2_O_2_ solution was prepared in phosphate buffer (pH 7.4). Then, 0.5 mL of each extract at different concentrations was added to the freshly prepared H_2_O_2_ solution and incubated at room temperature for 10 min. The absorbance of the reaction mixtures was measured at 230 nm against a blank solution.

#### 2.9.3 Total Antioxidant Capacity (TAC)

The total antioxidant capacity (TAC) of *B. alba* plant part extracts was determined using the phospho‐molybdenum method, as described by Marjanovic et al. (2021) with slight modifications. Briefly, 0.3 mL of each extract was mixed with 3 mL of reagent solution containing 0.6 M sulfuric acid, 28 mM sodium phosphate, and 4 mM ammonium molybdate. The reaction mixtures were incubated at 95°C for 90 min. After cooling to room temperature, the absorbance of each sample was measured at 695 nm. TAC values were expressed as milligrams of ascorbic acid equivalents per gram of dry extract (mg AAE/g dry extract).

### 2.10 Anti‐Diabetic Activity

The anti‐diabetic potential of the leaf, fruit, and stem extracts of *B. alba* was evaluated using two in vitro enzyme inhibitory assays, namely α‐amylase and α‐glucosidase inhibition assays.

#### 2.10.1 α‐Amylase Inhibition Assay

The α‐amylase inhibitory activity was determined following the method described by Nguelefack et al. (2020) with slight modifications. Briefly, 0.5 mL of each extract was mixed with 0.5 mL of α‐amylase solution (1 U/mL) and incubated at 25°C for 10 min. Subsequently, 0.5 mL of starch solution (1% w/v) was added as the substrate, and the mixture was incubated again at 25°C for 10 min. The reaction was terminated by adding 1 mL of 3, 5‐dinitrosalicylic acid (DNSA) reagent, followed by heating in a water bath at 95°C for 5 min. After cooling to room temperature (25°C), the mixture was diluted with 9 mL of distilled water, and absorbance was measured at 540 nm. The percentage of inhibition was calculated using the equation:

$$\%\text{of Inhibition}=\frac{\text{Absorbance of control}-\text{Absorbance of sample}}{\text{Absorbance of control}}\times 100$$

#### 2.10.2 α‐Glucosidase Inhibition Assay

The α‐glucosidase inhibitory activity was assessed according to the method described by Kidane et al. (2018). The reaction mixture consisted of maltose substrate, phosphate buffer (pH 6.8), and varying concentrations of the extract, incubated at 37°C. Afterwards, α‐glucosidase enzyme solution was added, and the mixture was further incubated at 37°C to initiate the reaction. The reaction was terminated by adding the DNSA reagent, and the tubes were heated at 95°C for 5 min. After cooling, absorbance was recorded at 540 nm. The percentage inhibition was calculated using the same formula as described above for the α‐amylase assay.

### 2.11 In Silico Studies

#### 2.11.1 Molecular Docking Study

##### 2.11.1.1 Selection and Preparation of Ligands

A total of fifteen compounds selected (five major compounds from each part) from the GC–MS results based on their higher peak area percentages and compliance with Lipinski's Rule of Five parameters, including molecular weight, log P, and the number of hydrogen bond donors and acceptors (Table 1). The selected molecules were retrieved in three‐dimensional (3D) SDF format (Hasan et al. 2025; Rahman et al. 2024) from the PubChem database. Before molecular docking (Hasan et al. 2025) all ligands were converted into 3D PDB format using Open Babel software and energy minimized via PyRx.

**TABLE 1:** Lipinski's rule‐based properties of selected bioactive compounds from leaf, fruit, and stem extracts of *B. alba* .

SI. No	Plant parts	Compounds name	Molecular weight (< 500KD)	Log *p* (< 5)	H‐bond donor (< 5)	H‐bond acceptor (< 10)
1	Leaf	1‐methyl‐5‐fluorouracil	144.10 g/mol	0.22	1	3
2		D‐alanine, N‐propargyloxycarbonyl‐, propargyl ester	206.28 g/mol	3.00	1	2
3		DL‐proline, 5‐oxo‐, methyl ester	143.14 g/mol	−0.06	1	3
4		Benzenacetaldehyde	120.15 g/mol	1.73	0	1
5		1H‐naphth[1,2‐d]imidazole	168.19 g/mol	2.39	1	1
6	Fruit	3‐methylquinoline	143.19 g/mol	2.43	0	1
7		2,4‐difluoro‐3‐hydroxybenzaldehyde	158.10 g/mol	1.75	1	4
8		5‐methoxypyrrolidin‐2‐one	115.13 g/mol	0.08	1	2
9		Vanillin	152.15 g/mol	1.20	1	3
10		Palmitoleic acid	254.41 g/mol	4.94	1	2
11	Stem	1‐ethyl‐1H‐pyrazole‐3,4‐diamine	126.16 g/mol	−0.18	2	1
12		5‐methyl‐2‐propan‐2‐yl‐1,3‐dioxan‐4‐one	158.19 g/mol	1.51	0	3
13		4,5‐dihydro‐3‐methyl‐1‐propyl‐1H‐pyrazole	126.20 g/mol	1.44	0	1
14		1,1‐diethyl‐4,4‐dimethyl‐2‐tetrazene	144.22 g/mol	1.40	0	2
15		3‐furancarboxylic acid, methyl ester	126.11 g/mol	1.06	0	3

##### 2.11.1.2 Preparation of Target Proteins

The crystal structures of human peroxiredoxin (PDB ID: 1HD2), oxidoreductase (PDB ID: 6NGJ), human pancreatic α‐amylase (PDB ID: 4 W93), and human maltase‐glucoamylase (PDB ID: 2QMJ) were retrieved in PDB format from the RCSB Protein Data Bank (https://www.rcsb.org/structure) for antioxidant and antidiabetic activity analyses. All protein structures were prepared by removing water molecules and heteroatoms using BIOVIA Discovery Studio 2025, followed by energy minimization in Swiss‐PdbViewer (Version 4.1.0). The minimized structures were then saved in PDB format for subsequent docking studies (Hasan et al. 2025; Rahman et al. 2024).

#### 2.11.2 Molecular Docking Analysis

Selected target proteins were docked with *B. alba* ligands using AutoDock Vina implemented within the PyRx 0.8 virtual screening environment (Dallakyan and Olson 2015; Trott and Olson 2010). A semi‐flexible docking approach was applied, in which protein structures were kept rigid while ligand conformations were treated as flexible. Grid boxes measuring 90 × 90 × 90 Å were configured at the active‐site centers of the respective proteins. The resulting docking poses and molecular interactions were visualized and analyzed using BIOVIA Discovery Studio Visualizer 2025 (Hasan et al. 2025).

#### 2.11.3 ADME/T Evaluation

The Absorption, Distribution, Metabolism, Excretion, and Toxicity (ADME/T) profiles of the selected bioactive compounds of *B. alba* were assessed using Lipinski's “rule of five” parameters through SwissADME and pkCSM, to evaluate their drug‐likeness and pharmacokinetic behavior (Daina et al. 2017; Pires et al. 2015).

### 2.12 Statistical Analysis

All experiments were performed in triplicate, and the results are presented as the mean ± standard deviation (SD). The IC_50_ values were determined using non‐linear regression analysis with GraphPad Prism software (version 10.0; GraphPad Software, San Diego, CA, USA). Statistical comparisons among groups were performed using one‐way analysis of variance (ANOVA), followed by Tukey's multiple comparison test. *p*‐value < 0.05 was considered statistically significant.

## 3 Results

### 3.1 Preliminary Phytochemical Screening

Qualitative phytochemical analysis of *B. alba* revealed the presence of alkaloids, flavonoids, phenols, tannins, and saponins in the leaf, fruit, and stem extracts. Steroids and terpenoids were detected in the leaf and fruit extracts, but were absent in the stem extract. The results of the qualitative phytochemical screening are summarized in Table 2.

**TABLE 2:** Preliminary qualitative phytochemical analysis of leaf, fruit, and stem extracts of *B. alba* .

Phytochemicals	Test	Observation		
		Leaf	Fruit	Stem
Alkaloids	Wagner test	+	+	+
	Mayer's test	+	+	+
	Hager's test	+	+	+
Flavonoids	Alkaline reagent test	+	+	+
	Shinoda test	+	+	+
Phenols	FeCl_3_ test	+	+	+
	Lead acetate test	+	+	+
Steroids	Libermann‐Burchardt test	+	+	−
Terpenoids	Salkowski test	+	+	−
Tannins	Bramers test	+	+	+
Saponins	Foam test	+	+	+

*Note:* +, present; −, absent.

### 3.2 Total Phenol and Flavonoid Content

**FIGURE 1:**
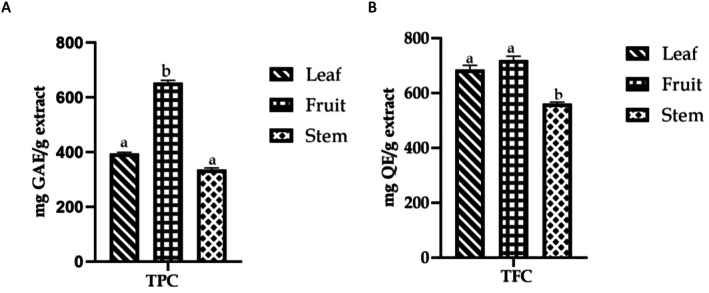
(A) Total phenolic content (TPC) and (B) total flavonoid content (TFC) of *B. alba* leaf, fruit, and stem extracts. Values are presented as mean ± standard deviation (SD). Different letters (a–b) indicate statistically significant differences among groups (*p* < 0.05, Tukey's test).

The total phenolic and flavonoid contents of *B. alba* plant organs were determined using standard colorimetric assays. The TPC values were 394.19 ± 4.60, 654.18 ± 7.97, and 335.97 ± 6.26 mg GAE/g for leaves, fruit, and stem, respectively (*p* < 0.05), indicating that the fruit extract contained the highest phenolic content compared to the other two parts. Similarly, the TFC values were 684.71 ± 16.46, 720.36 ± 13.99, and 561.29 ± 5.93 mg QE/g for leaves, fruit, and stem, respectively (*p* < 0.05), confirming that the fruit extract also contained the highest flavonoid content. A summary of the TPC and TFC values in the different *B. alba* plant parts are presented in Figure 1A,B.

### 3.3 FTIR Analysis

**FIGURE 2:**
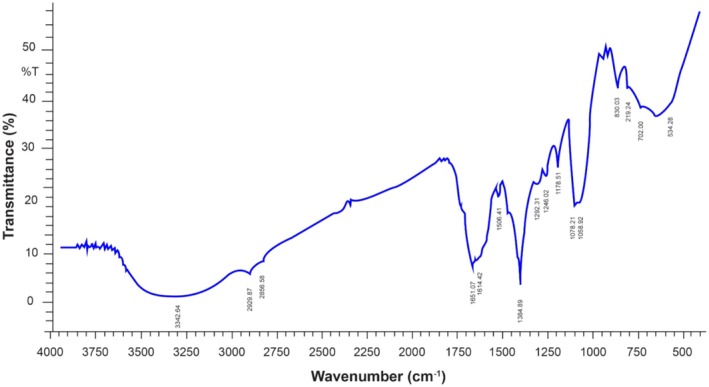
FT‐IR spectrum representing potential bands in the *B. alba* leaf extract.

**FIGURE 3:**
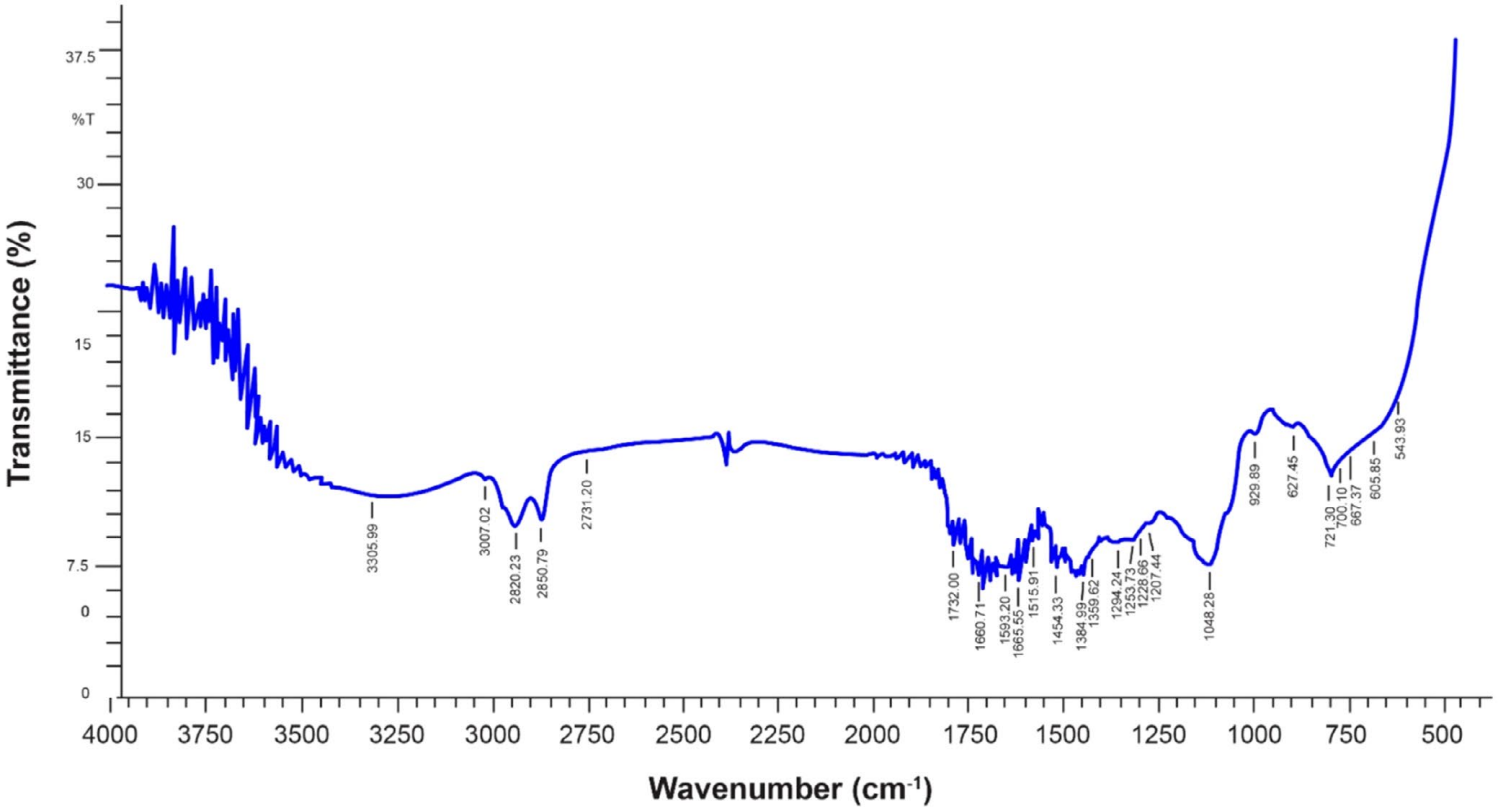
FT‐IR spectrum representing potential bands in the *B. alba* fruit extract.

**FIGURE 4:**
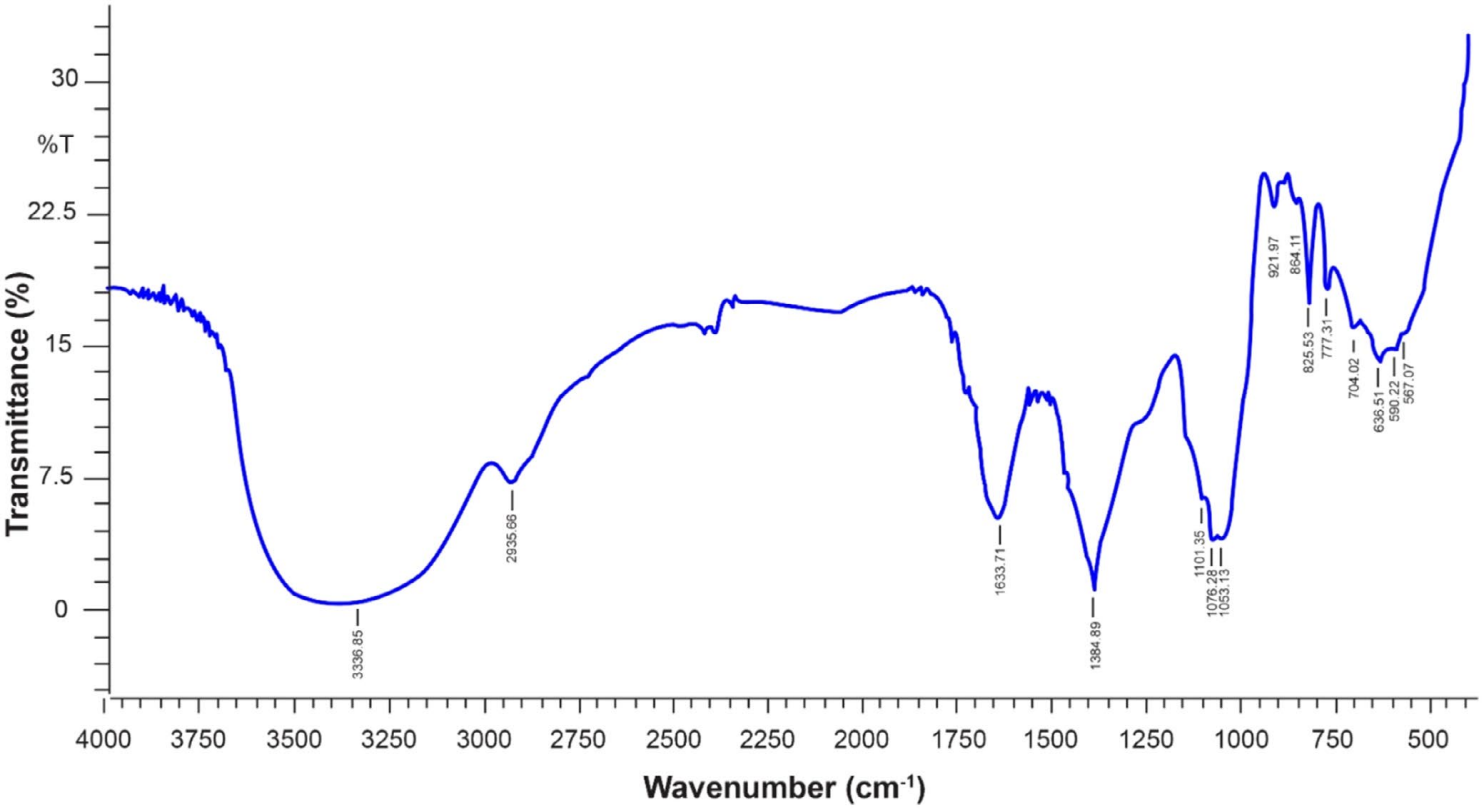
FT‐IR spectrum representing potential bands in the *B. alba* stem extract.

FTIR analysis of *B. alba* leaf, fruit, and stem extracts revealed the presence of multiple functional groups corresponding to diverse phytochemicals. All three plant parts exhibited broad O–H stretching vibrations, indicating the presence of alcohols and phenols, along with C–H stretching vibrations, confirming the presence of alkanes. Aromatic C=C stretching and out‐of‐plane C–H bending were also observed in all extracts, suggesting the presence of substituted aromatic compounds. The fruit extract displayed a prominent absorption band at 1732 cm^−1^ corresponding to C=O stretching, indicative of aldehydes, esters, or ketones. Additional peaks for C–N and C–O stretching confirmed the presence of alcohols, ethers, amines, and esters. Furthermore, minor halogenated compounds were detected in all extracts through C–Cl and C–Br stretching bands around 484–587 cm^−1^, with the stem extract showing particularly distinct signals. The results of FTIR analysis are summarized in Tables 3, 4, 5, and the corresponding FTIR spectra are presented in Figures 2, 3, 4, respectively.

**TABLE 3:** FTIR spectral wavenumber values, associated functional groups, and potential compound classes identified in *B. alba* leaf extract.

Wavenumber (cm^−1^)	Functional group	Possible compound class
~3342	O–H stretching (broad)	Alcohols, phenols
~2929, ~2856	C–H stretching (alkane)	Alkanes
~1651, ~1614	C=C stretching (aromatic)	Aromatic compounds
~1508	C=C stretching (possibly nitro)	Aromatic or nitro compounds
~1384	C–H bending	Alkanes
~1267, ~1243	C–N stretching	Amines
~1178, ~1078, ~1036	C–O stretching	Alcohols, ethers
~890, ~750, ~520	C–H bending, C–Br/C–Cl stretching	Aromatic or halogenated compounds

**TABLE 4:** Spectral wavenumber values, associated functional groups, and potential compound classes identified in *B. alba* fruit extract.

Wavenumber (cm^−1^)	Functional group	Possible compound class
3366	O–H stretching (broad)	Alcohols, phenols
2923, 2850	C–H stretching (alkane)	Alkanes
2731	C–H stretching (possibly aldehyde)	Aldehydes
1732	C=O stretching	Esters, aldehydes, ketones
1660, 1599, 1565	C=C stretching (aromatic), N–H bending	Aromatic compounds, amines
1510, 1444	Aromatic C–C or C=C stretching	Aromatic compounds
1384, 1338	C–H bending, NO_2_ symmetric stretch	Alkanes, nitro compounds
1310, 1264, 1250	C–N or C–O stretching	Amines, ethers
1207, 1162	C–O stretching	Alcohols, esters
1068	C–O, C–N stretching	Alcohols, amines
920, 827, 721	Aromatic C–H out‐of‐plane bending	Aromatic compounds
587, 543	C–Br, C–Cl stretching	Halogenated compounds

**TABLE 5:** FTIR spectral wavenumber values, associated functional groups, and potential compound classes identified in *B. alba* stem extract.

Wavenumber (cm^−1^)	Functional group	Possible compound class
3336.65	O–H stretching (broad)	Alcohols, phenols
2933.66	C–H stretching (alkane)	Alkanes
1633.71	C=C stretching (aromatic)	Aromatic compounds
1384.89	C–H bending (alkanes, methyl groups)	Alkanes, aliphatic groups
1267.02	C–N stretching	Aliphatic amines
1101.35	C–O stretching	Alcohols, esters, ethers
1029.13	C–O stretching	Alcohols, esters, ethers
823.53	Out‐of‐plane C–H bending	Aromatic compounds
773.31	Aromatic C–H bending	Aromatic compounds
704.02	Aromatic C–H bending	Aromatic compounds
654.51	C–Cl stretching	Alkyl halides
592.52	C–Cl stretching	Alkyl halides
547.07	C–Br stretching	Alkyl halides
521.07	C–Br stretching	Alkyl halides
484.11	C–Br stretching	Alkyl halides

### 3.4 GC–MS Analysis

**FIGURE 5:**
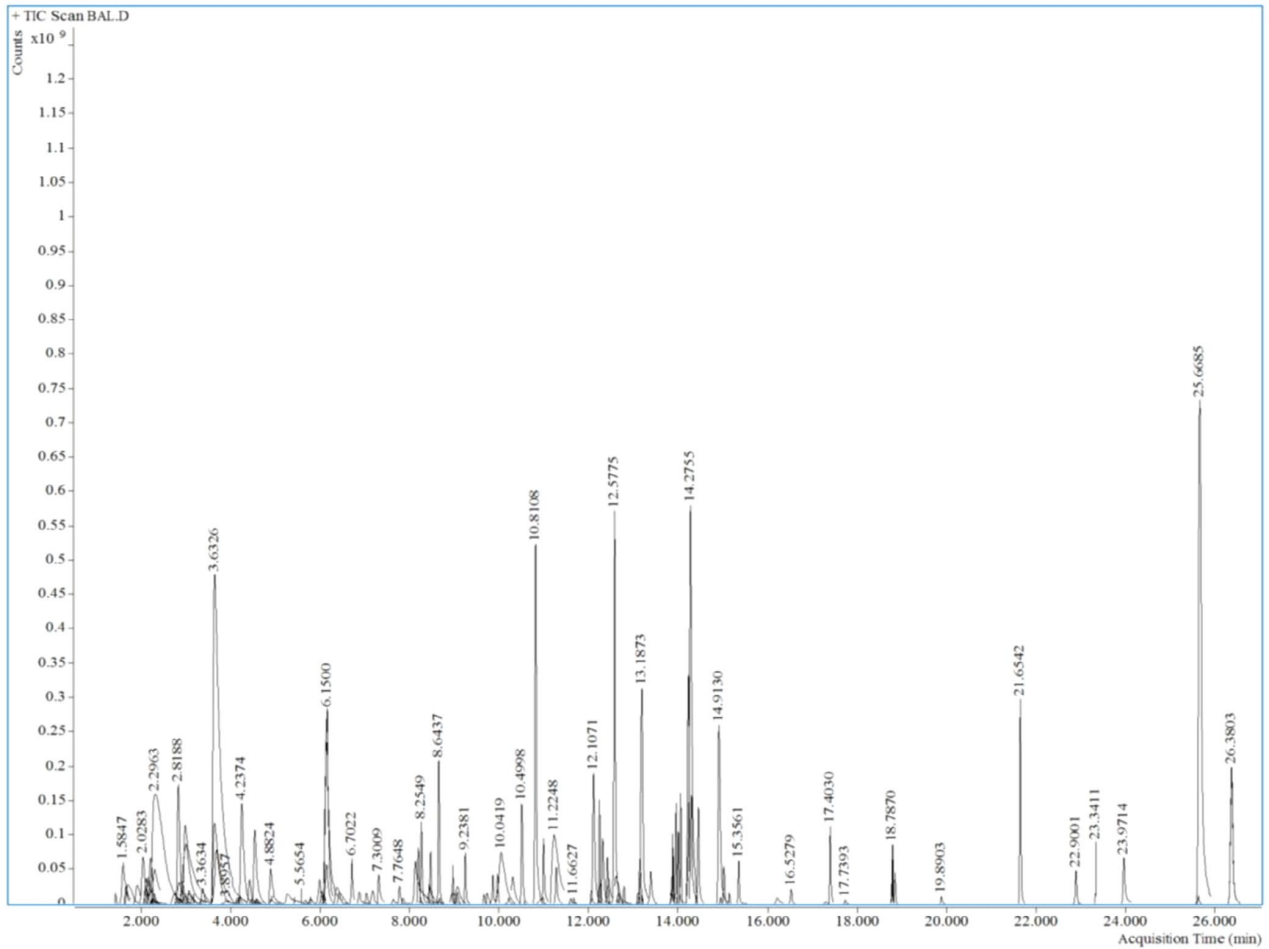
GC–MS total ion chromatogram of *B. alba* leaf extract.

**FIGURE 6:**
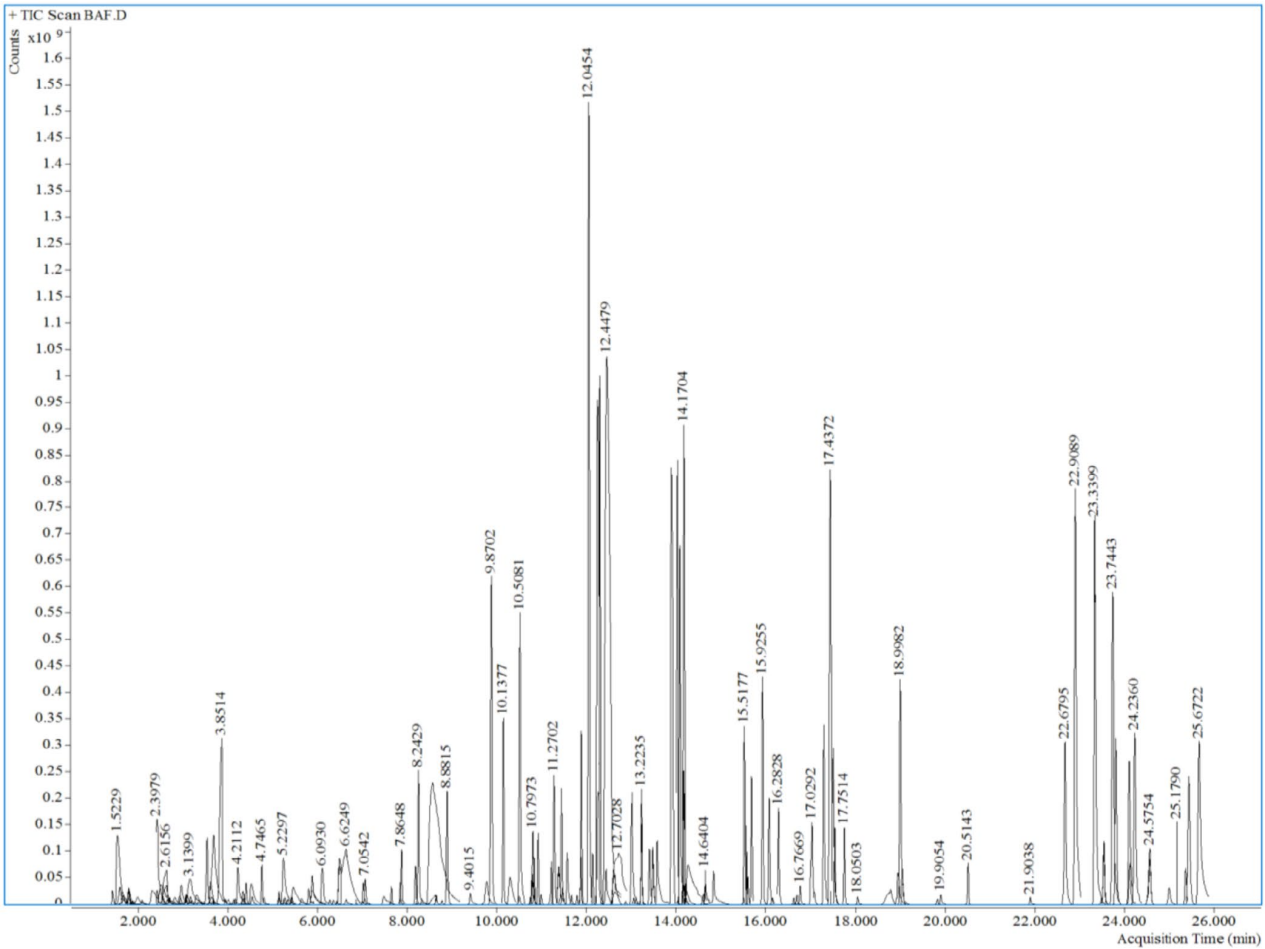
GC–MS total ion chromatogram of *B. alba* fruit extract.

**FIGURE 7:**
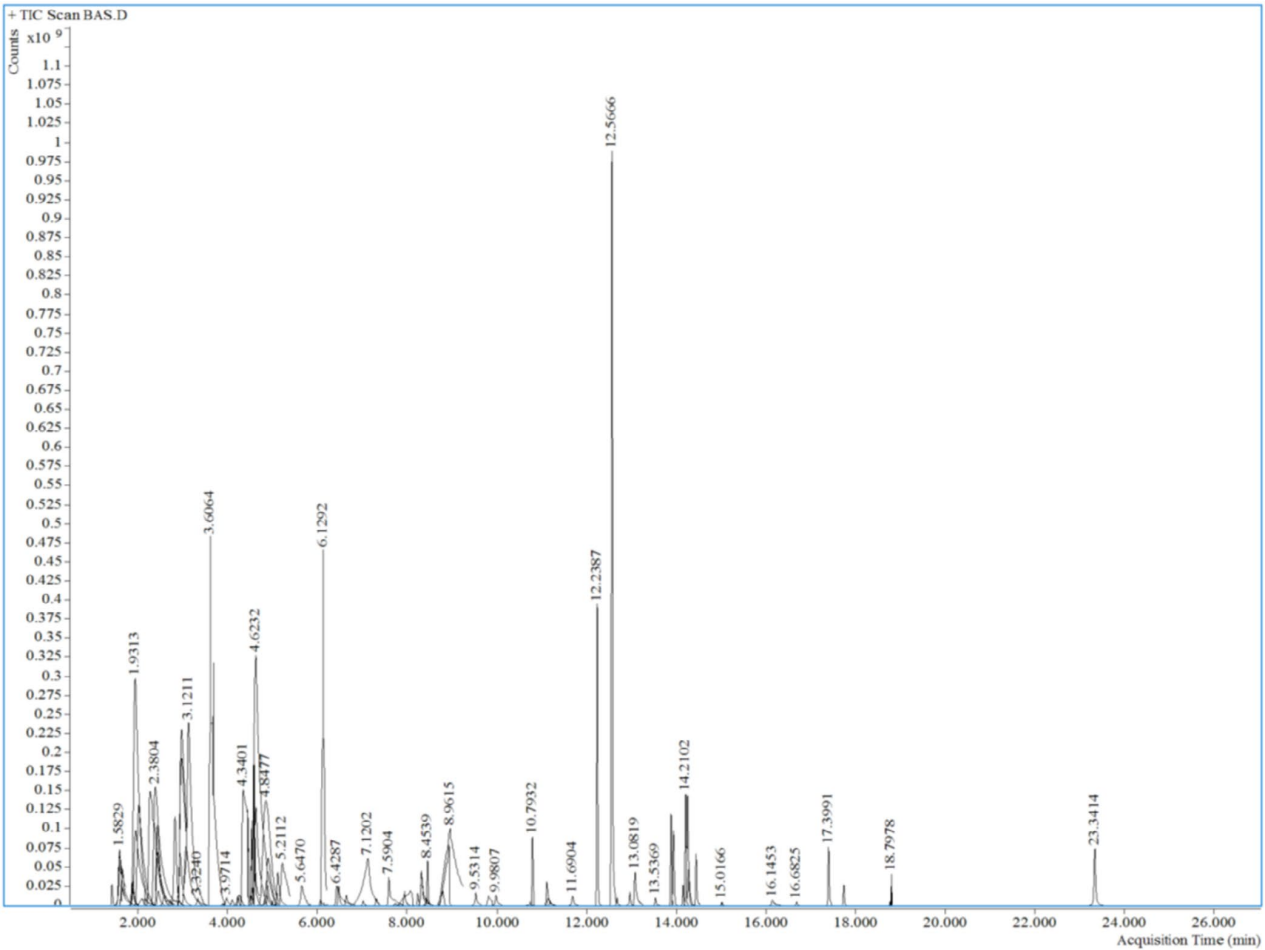
GC–MS total ion chromatogram of *B. alba* stem extract.

GC–MS analysis of *B. alba* identified diverse range of compounds in the leaf, fruit, and stem extracts, based on matching with the NIST20 library (350,704 EI mass spectra of 306,643 compounds). The common chemical compounds identified across the three organs of *B. alba* are summarized in Table 6. Among these, 30 major compounds with peak area percentages above 1%–2%, along with their retention times, molecular formulas, chemical classes, and molecular weights, are presented in Table 7 for the leaf, fruit, and stem extracts. The major constituents varied significantly among the plant organs. For instance, 1‐methyl‐5‐fluorouracil (9.63%) was identified as the predominant compound in the leaf extract, 3‐methyl‐quinolone (8.55%) in the fruit extract, and 1‐ethyl‐1H‐pyrazole‐3,4‐diamine (6.83%) in the stem extract. The total ion chromatograms (TICs) of the leaf, fruit, and stem extracts are shown in Figures 5, 6, 7, respectively.

**TABLE 6:** Phytochemical profiling of common bioactive compounds in leaf, fruit, and stem extracts of *B. alba* using GC–MS.

No.	RT	Compound name	Peak area in %		
			Leaf	Fruit	Stem
1	1.41	2,2‐difluoro‐ propane	0.112	0.263	0.211
2	1.59	Methyl formate	—	1.507	1.444
3	1.64	2‐pentanone	0.188	—	0.431
4	1.67	Pyrrolidine	—	0.17	0.091
5	1.9	1H‐1,2,4‐triazole‐5‐carbonitrile	0.897	0.046	—
6	1.97	Propanoic acid, 2‐oxo‐, methyl ester	—	0.361	2.74
7	2.82	Benzeneacetaldehyde	1.869	—	0.975
8	2.97	D‐alanine, N‐propargyloxycarbonyl‐, propargyl ester	7.094	0.774	4.804
9	3.06	3,5‐dimethyl‐1H‐pyrazole‐4‐carbaldehyde	0.376	—	1.675
10	3.16	5‐aminopyrimidine	0.315	0.673	—
11	3.63	1‐methyl‐5‐fluorouracil	9.631	0.364	—
12	3.67	(+)‐dibenzoyl‐L‐tartaric acid anhydride	1.23	1.828	—
13	3.97	3,6‐dimethyl‐1,2,4,5‐tetrazine	—	0.115	0.098
14	4.34	Niacin	—	0.028	0.081
15	4.41	3‐ethyl‐4‐methyl‐ 1H‐pyrrole‐2,5‐dione	0.396	0.143	—
16	4.52	Benzeneacetic acid	1.194	0.593	—
17	5.66	Benzamide	0.027	0.081	—
18	6.06	4‐nitro‐1H‐pyrazole	—	0.026	0.067
19	6.13	DL‐proline, 5‐oxo‐, methyl ester	5.916	1.102	3.464
20	6.36	4‐pyridinecarboxamide	0.451	0.039	0.329
21	6.48	vanillin	—	2.898	0.101
22	7.12	2‐amino‐2‐oxoethyl acetate	—	0.852	0.965
23	7.31	3‐phenyl‐pyridine	0.31	—	0.039
24	8.18	(R)‐4,4,7a‐trimethyl‐5,6,7,7a‐tetrahydrobenzofuran‐2 (4H)‐one	0.278	0.166	—
25	8.32	3‐hydroxy‐4‐methoxybenzoic acid	—	0.056	0.272
26	9.98	1,4‐dimethyl‐2 (1H)‐pyridinone	1.831	—	0.047
27	10.79	Loliolide	0.206	0.167	0.082
28	11.37	Adenine	2.789	0.779	—
29	11.66	Indole‐2‐carboxylic acid methyl ester	0.051	0.088	—
30	11.86	Hexadecanoic acid, methyl ester	0.229	0.117	0.447
31	12.57	n‐Hexadecanoic acid	0.606	—	0.59
32	13.18	1H‐naphth[1,2‐d]imidazole	2.129	—	0.223
33	13.88	9, 12‐Octadecadienoic acid (Z,Z)‐, methyl ester	0.041	—	0.054
34	14.08	Phytol	0.246	1.185	—
35	14.17	Methyl stearate		1.367	0.0356
36	14.21	(9E,11E)‐Octadecadienoic acid	0.379	—	0.083
37	14.25	9‐Octadecenoic acid (E)—	0.772	—	0.092
38	14.45	Octadecanoic acid	0.129	—	0.037
39	15.02	9H‐pyrido[3,4‐b]indole‐1‐carboxylic acid, methyl ester	0.133	—	0.005
40	17.5	Hexadecanoic acid, 2‐hydroxy‐1‐ (hydroxymethyl)ethyl ester	0.071	0.465	0.043
41	19.9	2,3‐diphenylquinoxaline	0.041	0.105	—
42	23.34	γ‐Sitosterol	0.013	0.511	0.032
43	25.66	Olean‐12‐en‐28‐oic acid, 3‐hydroxy‐, methyl ester (3b)—	2.839	1.232	—

**TABLE 7:** Major bioactive compounds identified by GC–MS in leaf, fruit, and stem extracts of *B. alba* .

Plant parts	SI No	RT	Class	Compound name	Formula	Molecular weight	Peak area %
Leaf	1	3.63	Pyrimidine derivative	1‐methyl‐5‐fluorouracil	C_5_H_5_FN_2_O_2_	144.03	9.63
	2	2.97	Amino acid derivative	D‐alanine, N‐propargyloxycarbonyl‐, propargyl ester	C_10_H_11_NO_4_	209.07	7.09
	3	6.13	Amino acid derivative	DL‐proline, 5‐oxo‐, methyl ester	C_6_H_9_NO_3_	143.05	5.91
	4	2.99	Lactam	2‐pyrrolidinone	C_4_H_7_NO	85.05	3.36
	5	25.66	Triterpenoid	Olean‐12‐en‐28‐oic acid, 3‐hydroxy‐methyl ester (3b)—	C_31_H_50_O_3_	470.37	2.83
	6	11.23	Purine alkaloid	Adenine	C_5_H_5_N_5_	135.05	2.78
	7	13.18	Imidazole derivative	1H‐naphth[1,2‐d]imidazole	C_11_H_8_N_2_	168.06	2.12
	8	2.82	Aldehyde	Benzeneacetaldehyde	C_8_H_8_O	120.058	1.86
	9	2.29	Nitrosamine	N‐nitrosodimethylamine	C_2_H_6_N_2_O	74.048	1.85
	10	10.04	Pyridinone derivative	2 (1H)‐pyridinone, 1,4‐dimethyl—	C_7_H_9_NO	123.068	1.83
Fruit	11	8.55	Quinoline alkaloid	3‐methylquinoline	C_10_H_9_N	143.07	8.55
	12	12.70	Aromatic aldehyde	2,4‐difluoro‐3‐hydroxybenzaldehyde	C_7_H_4_F_2_O_2_	158.02	5.41
	13	3.85	Lactam	5‐methoxypyrrolidin‐2‐one	C_5_H_9_NO_2_	115.06	3.37
	14	14.26	Heteroaromatic compound	Pyrrole	C_4_H_5_N	67.04	3.17
	15	6.63	Phenolic aldehyde	Vanillin	C_8_H_8_O_3_	152.05	2.892
	16	3.14	nitrogen heterocycle	4H‐1,2,4‐triazole, 4‐methyl—	C_3_H_5_N_3_	83.05	2.43
	17	12.44	Monounsaturated fatty acid	Palmitoleic acid	C_16_H_30_O_2_	254.23	2.06
	18	3.67	Acid anhydride	(+)‐dibenzoyl‐L‐tartaric acid anhydride	C_18_H_12_O_7_	340.06	1.83
	19	12.24	Fatty acid ester	Tridecanoic acid, methyl ester	C_14_H_28_O_2_	228.21	1.82
	20	23.74	Triterpenoid	β‐amyrin	C_30_H_50_O	426.39	1.75
Stem	21	4.44	Pyrazole derivative	1‐ethyl‐1H‐pyrazole‐3,4‐diamine	C_5_H_10_N_4_	126.09	6.83
	22	1.93	Furan derivative	furfural	C_5_H_4_O_2_	96.02	6.25
	23	4.62	Pyrazole derivative	4,5‐dihydro‐3‐methyl‐1‐propyl‐1H‐pyrazole	C_7_H_14_N_2_	126.11	5.98
	24	3.67	Tetrazene derivative	1,1‐diethyl‐4,4‐dimethyl‐2‐tetrazene	C_6_H_16_N_4_	144.14	5.73
	25	2.26	Alcohol	Glycerin	C_3_H_8_O_3_	92.045	5.33
	26	3.12	Furan derivative	3‐furancarboxylic acid, methyl ester	C_6_H_6_O_3_	126.03	4.56
	27	8.96	Lactone	5‐methyl‐2‐propan‐2‐yl‐1,3‐dioxan‐4‐one	C_8_H_14_O_3_	158.09	3.75
	28	6.12	Amino acid derivative	DL‐proline, 5‐oxo‐, methyl ester	C_6_H_9_NO_3_	143.06	3.46
	29	2.38	Pyrazole derivative	3‐methyl‐1H‐pyrazole‐4‐carbaldehyde	C_5_H_6_N_2_O	110.05	3.44
	30	2.01	Organic acid	Propanoic acid, 2‐oxo‐, methyl ester	C_4_H_6_O_3_	102.03	2.74

### 3.5 Antioxidant Activity

The antioxidant activity of *B. alba* extracts varied significantly across plant organs, as demonstrated by DPPH, hydrogen peroxide (H_2_O_2_) scavenging, and total antioxidant capacity (TAC) assays.

#### 3.5.1 DPPH Assay

**FIGURE 8:**
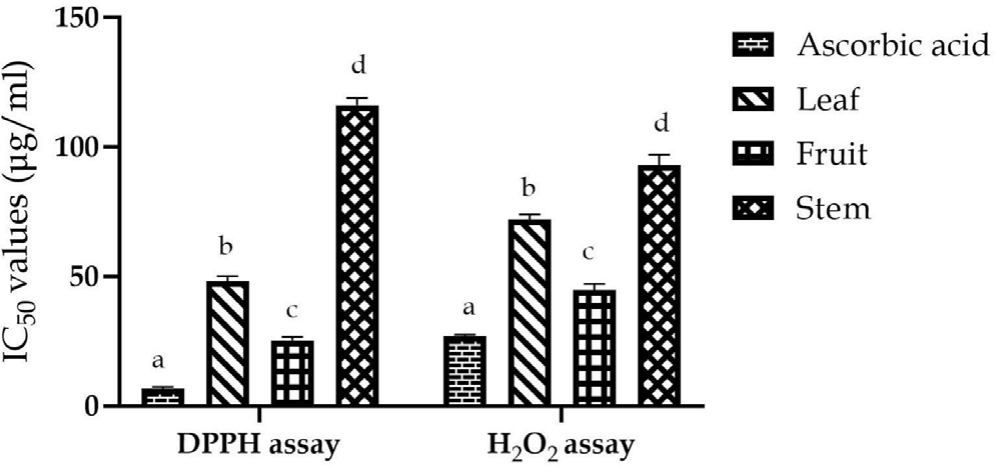
DPPH and H_2_O_2_ radical scavenging activities of *B. alba* leaf, fruit, and stem extracts expressed as IC_50_ values (mean ± SD). Different letters (a–d) indicate statistically significant differences among groups (*p* < 0.05, Tukey's test).

The free radical scavenging activity of *B. alba* leaf, stem, and fruit extracts was evaluated using the DPPH (2,2‐diphenyl‐1‐picrylhydrazyl) assay. The results indicated significant variation in antioxidant potential among the different plant parts. The fruit extract demonstrated the most potent radical scavenging activity, with an IC_50_ value of 25.27 ± 1.41 μg/mL. The leaf extract showed moderate activity, with an IC_50_ of 48.20 ± 1.96 μg/mL, while the stem extract exhibited the weakest activity (IC_50_ = 116.24 ± 2.74 μg/mL). For comparison, ascorbic acid, used as the standard antioxidant, displayed the highest radical scavenging activity with an IC_50_ of 6.74 ± 0.69 μg/mL (*p* < 0.05). The minimum inhibitory concentrations (IC_50_) for DPPH of *B. alba* plant parts are presented in Figure 8.

#### 3.5.2 Hydrogen Peroxide (H_2_O_2_ ) Scavenging Assay

The hydrogen peroxide (H_2_O_2_) scavenging activity of *B. alba* leaf, stem, and fruit extracts was evaluated to assess their antioxidant potential further. The results revealed that the fruit extract exhibited the highest H_2_O_2_ scavenging capacity with an IC_50_ value of 44.67 ± 2.54 μg/mL. The leaf extract showed moderate activity, with an IC_50_ of 71.90 ± 2.08 μg/mL, while the stem extract displayed the lowest activity (IC_50_ = 93.08 ± 3.99 μg/mL). Ascorbic acid, used as the standard, demonstrated the most potent scavenging activity with an IC_50_ value of 26.97 ± 0.55 μg/mL (*p* < 0.05), reflecting its well‐known strong antioxidant capacity. The minimum inhibitory concentrations (IC_50_) for H_2_O_2_ radical scavenging of *B. alba* plant parts are presented in Figure 8.

#### 3.5.3 Total Antioxidant Capacity (TAC)

**FIGURE 9:**
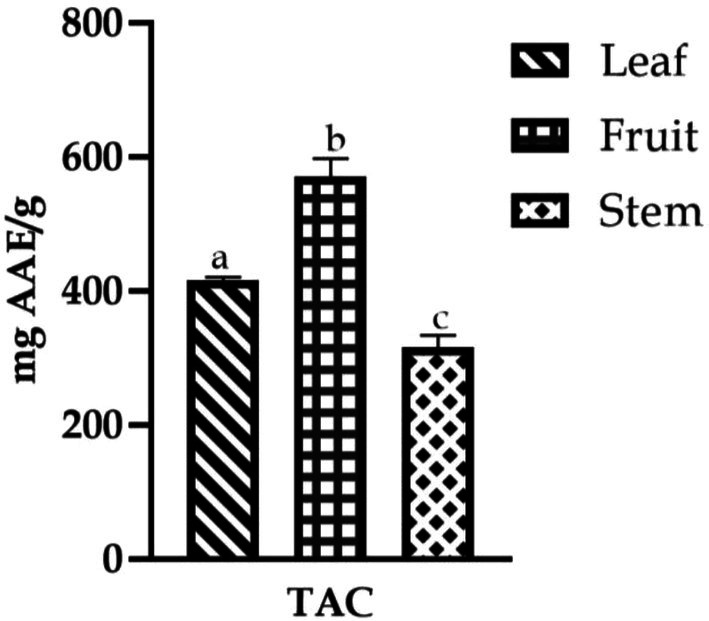
Total antioxidant capacity of *B. alba* leaf, fruit, and stem extracts (mean ± SD). Different letters (a–c) indicate statistically significant differences among groups (*p* < 0.05, Tukey's test).

To comprehensively evaluate the antioxidant potential of *B. alba* leaf, stem, and fruit extracts, the total antioxidant capacity (TAC) was assessed using the phosphomolybdenum method. The results were consistent with the trends observed in the DPPH and H_2_O_2_ scavenging assays. The fruit extract exhibited the highest TAC value of 570.09 ± 27.27 mg ascorbic acid equivalent per gram (mg AAE/g), indicating a strong capacity to reduce oxidants and neutralize reactive species. The leaf extract showed moderate activity with a TAC of 415.55 ± 18.18 mg AAE/g, whereas the stem extract displayed the lowest capacity (315.55 ± 18.18 mg AAE/g) (*p* < 0.05). The TAC values of the *B. alba* plant parts are presented in Figure 9.

### 3.6 Anti‐Diabetic Activity

**FIGURE 10:**
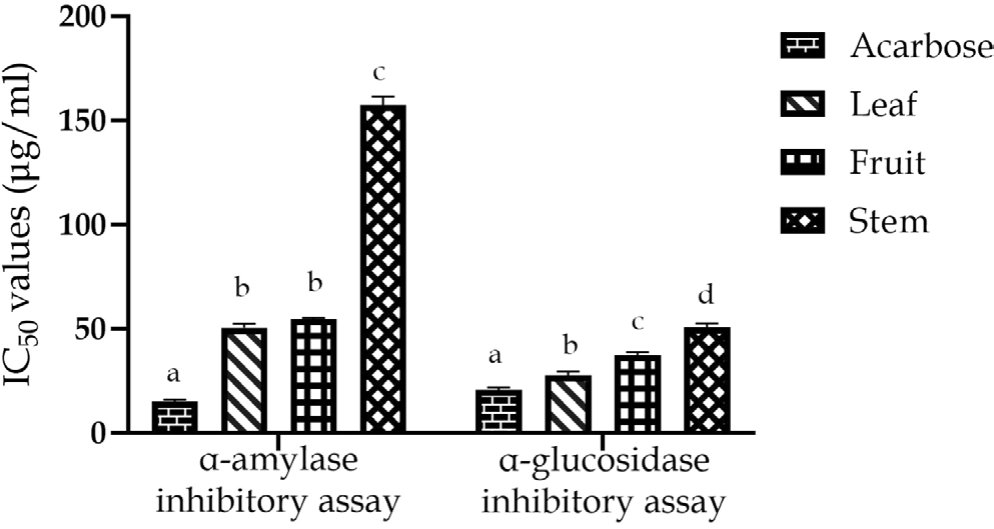
α‐Amylase and α‐glucosidase inhibitory activities of *B. alba* leaf, fruit, and stem extracts expressed as IC_50_ values (mean ± SD). Different letters (a–d) indicate statistically significant differences among groups (*p* < 0.05, Tukey's test).

The inhibitory potential of *B. alba* extracts against carbohydrate‐hydrolyzing enzymes varied significantly among the different plant parts. In the α‐amylase inhibition assay, the leaf extract exhibited the most potent inhibitory activity with an IC_50_ value of 50.63 ± 1.93 μg/mL, followed closely by the fruit extract (54.70 ± 0.77 μg/mL). In contrast, the stem extract showed the weakest inhibition (157.35 ± 4.48 μg/mL). The reference standard, acarbose, demonstrated the highest inhibitory activity with an IC_50_ value of 15.06 ± 0.94 μg/mL. Similarly, in the α‐glucosidase inhibition assay, the leaf extract showed the highest inhibitory effect, with an IC_50_ value of 27.81 ± 1.85, followed by the fruit extract (37.45 ± 1.43) and the stem extract (50.80 ± 1.87 μg/mL). Consistent with the α‐amylase assay, acarbose exhibited superior inhibition with an IC_50_ value of 20.77 ± 1.35 μg/mL (*p* < 0.05). The comparative data of IC_50_ values for α‐amylase and α‐glucosidase inhibitory activities are presented in Figure 10.

### 3.7 In Silico Study

#### 3.7.1 Molecular Docking Study

**FIGURE 11:**
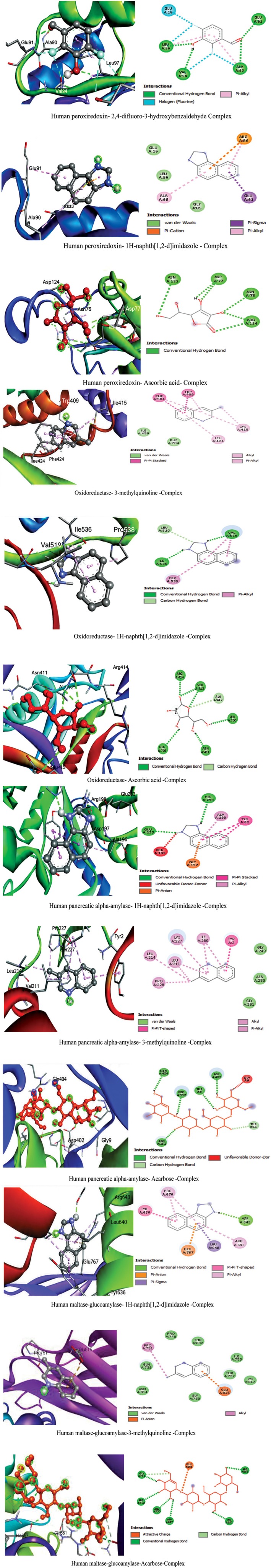
Molecular docking interactions of major GC–MS‐identified compounds from *Basella alba* leaf, stem, and fruit extracts, along with the reference standard, with target proteins: human peroxiredoxin (PDB ID: 1HD2), oxidoreductase (PDB ID: 6NGJ), human pancreatic α‐amylase (PDB ID: 4 W93), and human maltase‐glucoamylase (PDB ID: 2QMJ).

The molecular docking scores reflecting the binding affinity of each compound towards the respective proteins are presented in Table 8. It is found that 1H‐naphth[1,2‐d]imidazole from *B. alba* leaf showed highest antidiabetic potential by securing lowest binding energy (Table 8) against carbohydrate metabolizing enzymes. In contrast, 3‐methyl quinolone and 2,4‐difluoro‐3‐hydroxybenzaldehyde are the top two compounds from *B. alba* fruit that showed highest antioxidant properties with lowest binding energies (Table 8) against human peroxiredoxin and oxidoreductase target proteins. The binding affinity, interaction types, and binding residues of the top‐ranked docked compounds, along with the reference standards, are presented in Table 9 and illustrated in Figure 11.

**TABLE 8:** Molecular docking scores of major bioactive compounds identified by GC–MS in leaf, stem, and fruit extracts of *B. alba* against selected target proteins.

Plant parts	PubChem ID	Compound name	Binding energy (Kcal/mol)			
			1HD2	6NGJ	4W93	2QMJ
Leaf	225174	1H‐naphth[1,2‐d]imidazole	−5.2	−8.2	−6.6	−7.1
	500249	DL‐proline, 5‐oxo‐, methyl ester	−5.1	−5.3	−4.9	−4.6
	78957	1‐methyl‐5‐fluorouracil	−4.6	−5.1	−4.9	−5.6
	91712725	D‐alanine, N‐propargyloxycarbonyl‐, propargyl ester	−4.5	−5.7	−4.7	−5.5
	998	Benzenacetaldehyde	−4.4	−6.7	−5.0	−5.2
Fruit	11926	3‐methyl quinoline	−5.0	−8.3	−5.8	−6.3
	593909	2,4‐difluoro‐3‐hydroxybenzaldehyde	−5.4	−6.2	−5.5	−5.4
	1183	Vanillin	−4.9	−6.2	−5.2	−5.8
	445638	Palmitoleic acid	−4.3	−5.9	−4.9	−4.6
	181561	5‐methoxypyrrolidin‐2‐one	−4.4	−4.6	−3.9	−5.1
Stem	550065	5‐methyl‐2‐propan‐2‐yl‐1,3‐dioxan‐4‐one	−5.2	−5.6	−5.1	−5.0
	14918	3‐furancarboxylic acid, methyl ester	−4.4	−4.6	−4.2	−4.4
	566123	4,5‐dihydro‐3‐methyl‐1‐propyl‐1H‐pyrazole	−4.1	−4.9	−4.4	−4.2
	66509601	1‐ethyl‐1H‐pyrazole‐3,4‐diamine	−4.3	−4.9	−5.0	−4.6
	541943	1,1‐diethyl‐4,4‐dimethyl‐2‐tetrazene	−3.9	−4.3	−4.1	−4.2
Standards	54670067	Ascorbic acid	−5.4	−6.1	—	—
	86583399	Acarbose	—	—	−7.9	−7.7

**TABLE 9:** Molecular interaction profile illustrating bond types and interacting amino acid residues of major GC–MS‐identified bioactive compounds from *B. alba* leaf, fruit, and stem extracts and the standard compound with selected target proteins.

Protein	PubChem ID	Compound name	Binding energy (Kcal/mol)	Hydrogen bond		Hydrophobic bond			Electrostatic bond		
				Conventional	Carbon‐hydrogen	Pi‐alkyl	Alkyl	Others	Pi‐Cation	Pi‐Anion	Others
1HD2	593909	2,4‐difluoro‐3‐hydroxybenzaldehyde	−5.4	VAL94, ALA90, GLU91, LEU96	_	ALA90, LEU96	_	_	_	_	Halogen: GLU16, ALA90, VAL94, LEU96
	225174	1H‐naphth[1,2‐d]imidazole	−5.2	_	_	ARG86, ALA90	_	Pi‐Sigma: GLU91	ARG86	_	_
	54670067	Ascorbic acid	−5.4	ASN76, ASN122, ASP77, ARG124		_	_	_		_	
6NGJ	11926	3‐methyl quinoline	−8.3	_	_	TRP409, PHE584, LEU424	CYS415	Pi‐Pi Stacked: TRP409, PHE584	_	_	_
	225174	1H‐naphth[1,2‐d]imidazole	−8.2	VAL519, ILE536	LEU520	PRO538, VAL519		_	_	_	_
	54670067	Ascorbic acid	−6.1	SER413, ARG414	_	_	_	_	_	_	_
4 W93	225174	1H‐naphth[1,2‐d]imidazole	−6.6	HIS299, GLU233	_	ALA198	_	Pi‐Pi Stacked: TYR62	_	ASP197	
	11926	3‐methyl quinoline	−5.8	_	_	ILE230	LEU211, LEU214, LYS227, PRO228	Pi‐Pi T‐shaped: TYR2	_	_	_
	86583399	Acarbose	−7.9	PRO332, ASP402, GLN404, THR6, ARG252	THR11		_	_	_	_	_
2QMJ	225174	1H‐naphth[1,2‐d]imidazole	−7.1	ASP649	_	ARG643, PRO676		Pi‐Sigma: LEU640 Pi‐Pi T‐shaped: TYR636	_	GLU767	_
	11926	3‐methyl quinoline	−6.3	_	_	_	PRO751	_	_	GLU815	_
	86583399	Acarbose	−7.7	GLY731, ARG712, ARG730, ASP697, HIS657	GLY731		_	_	_	_	_

#### 3.7.2 ADME/T Properties

The drug‐likeness of the *B. alba* compounds was evaluated using Lipinski's “rule of five”, and all evaluated phytoconstituents were found to comply with the criteria for favorable oral bioavailability. Comprehensive ADME/T profiling of the selected compounds is summarized in Table 10.

**TABLE 10:** In silico ADMET analysis of selected major bioactive compounds identified by GC–MS in *B. alba* leaf, fruit, and stem extracts.

Plant parts	Name of compounds	Absorption		Distribution		Metabolism	Excretion	Toxicity		Drug likeliness	Bioavailability
		Water solubility (log mol/L)	Intestinal absorption (%)	VDss (human) (log L/kg)	BBB permeability (log BB)	CYP3A4 substrate	Total clearance (log ml/min/kg)	AMES toxicity	Hepatotoxicity		
Leaf	1H‐naphth[1,2‐d]imidazole	−2.89	77.639	−0.14	0.741	No	0.931	No	No	Yes	0.55
	DL‐proline, 5‐oxo‐, methyl ester	−0.103	91.673	−0.172	−0.291	No	0.657	No	No	Yes	0.55
	1‐methyl‐5‐fluorouracil	−0.639	92.71	−0.375	−0.307	No	0.626	No	No	Yes	0.55
	D‐alanine, N‐propargyloxycarbonyl‐, propargyl ester	−0.382	89.193	−0.3	−0.233	No	0.804	No	No	Yes	0.55
	Benzenacetaldehyde	−1.508	95.899	0.145	0.158	No	0.331	No	No	Yes	0.55
Fruit	3‐methyl quinoline	−2.232	96.877	−0.072	0.354	No	0.275	No	No	Yes	0.55
	2,4‐difluoro‐3‐hydroxybenzaldehyde	−0.98	91.645	−0.252	−0.217	No	0.367	No	No	Yes	0.55
	Vanillin	−1.308	84.976	−0.152	−0.243	No	0.601	No	No	Yes	0.55
	Palmitoleic acid	−5.477	92.51	−0.574	−0.084	Yes	1.817	No	No	Yes	0.85
	5‐methoxypyrrolidin‐2‐one	0.062	94.222	−0.079	−0.26	No	0.608	No	No	Yes	0.55
Stem	5‐methyl‐2‐propan‐2‐yl‐1,3‐dioxan‐4‐one	−1.277	100	−0.032	−0.14	No	0.275	No	No	Yes	0.55
	3‐furancarboxylic acid, methyl ester	−0.724	99.485	−0.259	−0.259	No	0.78	No	No	Yes	0.55
	4,5‐dihydro‐3‐methyl‐1‐propyl‐1H‐pyrazole	−1.868	97.381	0.194	0.116	No	0.595	No	No	Yes	0.55
	1‐ethyl‐1H‐pyrazole‐3,4‐diamine	−1.648	94.96	−0.072	0.089	No	0.347	No	No	Yes	0.55
	1,1‐diethyl‐4,4‐dimethyl‐2‐tetrazene	−1.694	100	−0.044	0.133	No	0.848	No	No	Yes	0.55

## 4 Discussion

Phytochemicals are naturally occurring bioactive compounds in plants that contribute to human health by reducing the risk of chronic diseases. They exhibit diverse biological activities, including antioxidant, anti‐inflammatory, anticancer, antidiabetic, cardioprotective, and neuroprotective effects (Debnath et al. 2021; Panche et al. 2016). The phytochemical screening of *B. alba* plant parts revealed a diverse range of secondary metabolites, including flavonoids, phenols, alkaloids, terpenoids, saponins, tannins, and steroids (Table 2). The presence of these phytoconstituents highlights the therapeutic potential of *B. alba* and supports previous findings regarding its ethnomedicinal applications in managing inflammation, diabetes, and oxidative stress‐related disorders (Dahanayaka et al. 2025; Tongco et al. 2015).

The total phenol and flavonoid estimation for all three parts of *B. alba* revealed highest phenol and flavonoid content in fruit extract compared to leaf and stem extract indicating the order fruit > leaf > stem. As most of the previous studies focused on *B. alba* leaf extract in TPC and TFC estimation (Halayal et al. 2025; Kumar et al. 2023) the present study provides a comparative evaluation of total polyphenols and flavonoids across *B. alba* leaf, fruit and stem. Previous studies revealed a wide variation in that phytochemicals content across different geographic regions (Dahanayaka et al. 2025) and these results are consistent with our findings. The elevated concentrations of phenols and flavonoids in fruit extracts suggest its stronger antioxidant properties and therapeutic efficacy, particularly against oxidative stress‐related disorders (Chen et al. 2022; Zhang et al. 2019).

The Fourier Transform Infrared (FTIR) spectroscopy analysis of *B. alba* leaf, stem, and fruit extracts demonstrated the presence of a diverse array of functional groups, reflecting the complex phytochemical composition of the plant. Many previous studies have already done FTIR analysis for *B. alba* leaf or fruit dye or for another species known as *Basella rubra* in order to predict phyto‐compounds of pharmacological importance (Gokilamani et al. 2015; Lingeshwaran et al. 2024). Therefore, the present study highlights a comparative analysis of FTIR spectrum of three different parts of *B. alba* , that is, leaf, fruit and stem which will facilitate further identification of diverse phytochemicals in different analysis like GC–MS and strengthen the obtained results. A broad absorption band observed around 3340–3366 cm^−1^ in all extracts corresponded to O–H stretching vibrations, characteristic of alcohols and phenolic compounds. These groups are widely recognized for their antioxidant and free radical–scavenging activities (Nur et al. 2023; Zhang, Shuai, et al. 2024). The C–H stretching vibrations detected in all parts between 2920 and 2850 cm^−1^ indicated the presence of alkane groups, typically associated with long‐chain fatty acids and hydrocarbons that may contribute to membrane stabilization and antimicrobial properties (Wongsa et al. 2022). Distinct C=C stretching bands in the range of 1614–1660 cm^−1^ confirmed the presence of aromatic systems, which are commonly found in polyphenolic structures such as flavonoids and phenolic acids (Pasieczna‐Patkowska et al. 2025). Interestingly, the fruit extract exhibited an additional strong band at 1732 cm^−1^, corresponding to C=O stretching vibrations of aldehydes, ketones, or esters. These carbonyl‐containing compounds are often derived from lipid peroxidation or secondary metabolism and are known for their antioxidant and antimicrobial properties (Sanou et al. 2024). This observation is consistent with the superior antioxidant and enzyme inhibitory activities demonstrated by the fruit extract in the present study.

Furthermore, absorption peaks detected in the 1260–1310 cm^−1^ regions in both leaf and fruit extracts indicated C–N and C–O stretching vibrations, suggesting the presence of amines and ethers (Pasieczna‐Patkowska et al. 2025). These groups are typically associated with alkaloids and glycosides, which contribute to enzyme inhibition and anti‐inflammatory effects (Kritsi et al. 2022). The leaf and stem extracts exhibited absorption below 600 cm^−1^, corresponding to C–Cl and C–Br stretching vibrations, indicative of alkyl halide‐like compounds. Additionally, the fruit extract displayed a peak at 1338 cm^−1^, corresponding to the N–O symmetric stretching of nitro compounds, which are reported to possess antioxidant and cytotoxic properties (Imon et al. 2023). Therefore, the FTIR spectral profile confirms the chemical diversity of *B. alba* and highlights the presence of functionally important phyto‐constituents.

GC–MS revealed bioactive metabolites including amino acid derivatives, pyrimidines, triterpenoids, and heterocycles. Major compounds identified in *Basella alba* leaf included 1‐methyl‐5‐fluorouracil (9.63%), a pyrimidine analogue with antioxidant and cytotoxic potential (Hadi et al. 2024), DL‐proline methyl ester (5.92%) and D‐alanine propargyl ester (7.09%), both linked to antioxidant and enzyme inhibitory activities (Wu 2020). 2‐pyrrolidinone (3.36%) and olean‐12‐en‐28‐oic acid, 3‐hydroxy‐methyl ester (2.84%) further contribute to antioxidant and anti‐inflammatory effects (Kairytė et al. 2022; Triaa et al. 2024). Compounds belonging to heterocyclic or imidazole‐type classes such as those analogous to 1H‐naphth[1,2‐d]imidazole (2.12%) have been documented to exert antimicrobial and anticancer activities (Mahmood et al. 2023; Ahmad et al. 2023). Key compounds identified from *B. alba* fruit included 3‐methylquinoline (8.55%) with antioxidant and antidiabetic relevance (Hernández‐Ayala et al. 2023; Kumar et al. 2025), 2,4‐difluoro‐3‐hydroxybenzaldehyde (5.41%) with radical scavenging activity (Li et al. 2023), 5‐methoxypyrrolidin‐2‐one (pterolactam, 3.37%) with anti‐inflammatory potential (Dascalu et al. 2020), pyrrole derivatives (3.17%) supporting antidiabetic activity (Bhat et al. 2024), and vanillin (2.89%) promoting insulin sensitivity and α‐glucosidase inhibition (Salau et al. 2024; Bhandari et al. 2021). Palmitoleic acid (2.06%) also enhances glucose metabolism and reduces inflammation (Guo et al. 2022; Liang et al. 2024). Predominant metabolites from *B. alba* stem include 1‐ethyl‐1H‐pyrazole‐3,4‐diamine (6.83%) with antifungal and antioxidant effects (Yadav et al. 2025), furfural (6.25%) and 3‐furancarboxylic acid, methyl ester (4.56%) contributing to radical scavenging and carbohydrate enzyme inhibition (Emil et al. 2024; Nguena‐Dongue et al. 2023), and 4,5‐dihydro‐3‐methyl‐1‐propyl‐1H‐pyrazole (5.98%) supporting antioxidant, anti‐inflammatory, and antidiabetic effects (Dhouib et al. 2023). Glycerin (5.33%) aids antioxidant and glucose‐regulating activities (Li et al. 2025; Kowalska et al. 2021). This comparative phytochemical investigation revealed that the three plant parts share several core metabolites (Table 6) but also exhibit unique major constituents (Table 7) that may contribute to distinct biological functions. These findings are consistent with previous GC–MS studies of *B. alba* that identified metabolites, including fatty acids, sterols, and small heterocyclic compounds (Rahman et al. 2021; Siju and Krishnakumar 2022). Considering that most previous studies have focused on single plant parts (Baskaran et al. 2015; Nur et al. 2023; Omotoso et al. 2024), the present study provides the first comparative phytochemical analysis of leaves, fruits, and stems of *B. alba* , highlighting its broad pharmacological potential. Hence, the presence of these phytochemicals in the leaf, stem, and fruit extracts of *B. alba* likely underpins their potent therapeutic effects, including antioxidant and antidiabetic activities. These findings align with recent studies highlighting the ethnomedicinal uses of *B. alba* in managing oxidative stress, inflammation, and diabetes, suggesting its potential as a natural source for bioactive compounds in nutraceutical and pharmaceutical applications (Halayal et al. 2025; Nur et al. 2023).

DPPH, H_2_O_2_ free radical scavenging activity as well as total antioxidant capacity (TAC) assessment again revealed that *B. alba* fruit extract had a higher antioxidant potential compared to leaf and stem parts. A number of previous investigations determined antioxidant potential of *B. alba* but they primarily focused on the leaf or a single plant part (Dahanayaka et al. 2025; Halayal et al. 2025; Sheik et al. 2023) Therefore, the present study demonstrates a comparative evaluation of antioxidant potential across three different aerial parts of *B. alba* . The consistent pattern across all antioxidant assays: fruit > leaf > stem indicates that *B. alba* fruit extract possesses the most potent antioxidant activity. This may be associated with its elevated levels of phenolic and flavonoid compounds, which play pivotal roles in neutralizing ROS, preventing lipid peroxidation, and enhancing cellular antioxidant defenses (Das et al. 2024).

It is already established that *B. alba* leaves have hypoglycemic properties through different in vivo former investigations (Arokoyo et al. 2018; Bamidele et al. 2025). Our study, therefore, was done to assess hypoglycemic potential of other aerial parts too, that is, fruit and stem along with leaves. In vitro α‐amylase and α‐glucosidase enzyme inhibition assays revealed *Basella alba* leaf extract had higher anti‐diabetic properties with lowest IC_50_ values compared to fruit and stem parts. These findings are consistent with previous studies that have reported significant in vitro carbohydrate metabolizing enzyme inhibitory activity in *B. alba* leaf extracts (Dedvisitsakul and Watla‐Iad 2022; Febriyanti et al. 2025). The observed inhibitory activities may be attributed to the presence of phytochemicals, for example, polyphenolic compounds in *B. alba* , which are known to inhibit digestive enzymes by binding to active or allosteric sites, thereby altering enzymatic function (Ćorković et al. 2022). As previous studies only focused on *B. alba* leaf anti‐diabetic potential, the present study demonstrates a comparative overview of hypoglycemic property across different aerial parts of this plant and confirms its highest hypoglycemic potentiality in leaves.

Previous *in silico* studies have mostly done for *Basella alba* leaf or any single part of this plant and outlined anti‐inflammatory, anti‐cancer, tumor suppressor activities of respective compounds (Halayal et al. 2025; Nur et al. 2023; Oktavriana et al. 2025). As none of those studies specifically targeted the antioxidant and antidiabetic activities of phytochemicals identified in GC‐ MS analysis, our study focuses on this respect and again provides a comparative evaluation of docking results across the plant parts. The molecular docking analysis of *B. alba* phytochemicals demonstrated strong binding affinities towards target proteins, often comparable to or exceeding those of the standard reference compounds. Notably, 1H‐naphth[1,2‐d]imidazole from leaf extract and 3‐methyl quinolone as well as 2,4‐difluoro‐3‐hydroxybenzaldehyde from fruit extract showed higher binding affinities against peroxiredoxin (1HD2) and oxidoreductase (6NGJ) than the standard antioxidant ascorbic acid which reveals their excellent antioxidant potential. Upon comparison from the obtained results, it can be said that compounds from the fruit extract possess highest antioxidant potential and can be used as lead compound to design drugs that can prevent oxidative stress and related disorders. In terms of anti‐diabetic potential, 1H‐naphth[1,2‐d]imidazole demonstrated the strongest binding affinity towards α‐amylase (4W93) and maltase‐glucoamylase (2QMJ), with docking scores near to the standard inhibitor acarbose. These findings make 1H‐naphth[1,2‐d]imidazole a potential lead compound for designing a new and better blood glucose controlling agent. Conversely, compounds from the *B. alba* stem extract exhibited comparatively lower binding affinities, indicating moderate antioxidant and anti‐diabetic capacity. Key molecular interactions, for example, hydrogen bonds, hydrophobic contacts, and the amino acid residues involved are listed in Table 9. Figure 8 further illustrates the interaction profiles of the top‐ranked compounds within the active site pockets. Drug‐likeness evaluation using Lipinski's “rule of five” confirmed that all analyzed *B. alba* phytochemicals met the criteria for favorable oral bioavailability. Lipinski‐compliant physicochemical properties are widely regarded as predictive of good pharmacokinetic performance in early drug screening (Lipinski et al. 2001). Furthermore, the ADME/T profiling (Table 10) indicated acceptable absorption and toxicity properties, which are comparable to those predicted by computational models and predictive tools such as SwissADME, commonly used in phytochemical drug discovery (Daina et al. 2017). Recent ADME/T modeling studies emphasize the value of such analyses for identifying phytochemicals with lead‐like potential (Alagarsamy et al. 2024; Cherian and Vadivel 2023). These findings suggest that *B. alba* differentaerial parts are promising sources of bioactive molecules with potent antioxidant and anti‐diabetic potential, supported by both in vitro assays and computational validation. However, the study is limited to in vitro analyses and molecular docking; further in vivo and clinical studies are needed to validate these findings.

## 5 Conclusion

The present study provides a comprehensive evaluation of the phytochemical composition and bioactivity of different parts of *B. alba* , integrating spectroscopic, chromatographic, and in vitro analyses. FTIR spectroscopy revealed the presence of functional groups characteristic of flavonoids, phenolics, carboxylic acids, alkanes, alcohols, and other bioactive constituents. GC–MS profiling further identified a diverse range of phytochemicals which are known for their antioxidant, anti‐diabetic, and other bioactivities. Quantitative phytochemical analysis revealed that the fruit contained the highest levels of phenolics and flavonoids, which correlated strongly with its superior antioxidant performance in DPPH radical scavenging, hydrogen peroxide scavenging, and total antioxidant capacity assays. In contrast, the leaf extract exhibited the most potent inhibitory activity against α‐amylase and α‐glucosidase, indicating its potential for postprandial glycemic regulation. These findings validate the ethno‐medicinal use of *B. alba* and demonstrate that different parts offer distinct pharmacological benefits, with fruits serving as a rich source of antioxidants and leaves acting as a potent anti‐diabetic resource as well as stems having moderate antioxidant and hypoglycemic properties. The integration of FTIR and GC–MS data with in vitro bioassays provides a robust scientific basis for utilizing *B. alba* in nutraceuticals and functional foods. The promising results establish a foundation for further exploration of *B. alba* in the prevention and management of oxidative stress, diabetes and related disorders. The observed differential bioactivity among plant parts underscores the importance of targeted use depending on the desired therapeutic outcome. Future studies should focus on the isolation and characterization of bioactive compounds, as well as in vivo validation, to advance the therapeutic application of this plant.

## Author Contributions

**Shorna Das:** methodology, software, data curation, investigation, validation, writing – original draft. **Bidduth Kumar Sarkar:** visualization, writing – original draft. **Sukalyan Kumar Kundu:** writing – review and editing, visualization, resources. **Pradip Debnath:** conceptualization, resources, supervision, software, writing – review and editing. **Satyajit Roy Rony:** methodology, software, data curation, investigation, formal analysis, writing – original draft.

## Ethics Statement

The authors have nothing to report.

## Conflicts of Interest

The authors declare no conflicts of interest.

## Data Availability

The data supporting the findings of this study can be obtained from the corresponding author upon reasonable request.
